# Serosurvey for Infectious Agents Associated with Subfertility and Abortion in Dairy Cattle in Trinidad and Tobago, West Indies

**DOI:** 10.3390/vetsci5020051

**Published:** 2018-05-11

**Authors:** Michael J. Morris, Jamie Sookhoo, Lemar Blake, Arianne Brown Jordan, Justine John, Sheliza Ali, Gervaise Sarjusingh, Janelle St. Aime, Edward H. Amoroso, Christopher A. L. Oura

**Affiliations:** 1 School of Veterinary Medicine, The University of the West Indies (St. Augustine), Eric Williams Medical Sciences Complex, Mount Hope, Trinidad and Tobago; jvekesh@hotmail.com (J.S.); lemar.blake@sta.uwi.edu (L.B.); brown.arianne@gmail.com (A.B.J.); justinejohn1991@gmail.com (J.J.); priyanka.hope.ali@gmail.com (S.A.); gervaisegs@gmail.com (G.S.); cbnellz24@gmail.com (J.S.A.); chris.oura@sta.uwi.edu (C.A.L.O.); 2 Nestlé, Churchill Roosevelt Highway, Trinidad and Tobago; Edward.amoroso01@gmail.com

**Keywords:** Trinidad and Tobago, ELISA, *Brucella abortus*, *Neospora caninum*, Bovine Viral Diarrhoea virus (BVDV), Infectious Bovine Rhinotracheitis virus (IBRV)

## Abstract

Despite frequent reports of subfertility and abortion in dairy cattle in Trinidad and Tobago (T&T), little is known about the potential infectious and non-infectious causes. This study set out to investigate possible infectious causes of reproductive problems by measuring the seroprevalence of four of the most significant reproductive pathogens in dairy cattle worldwide: *Brucella abortus (B. abortus)*; *Neospora caninum* (*N. caninum*), Bovine Viral Diarrhoea virus (BVDV), and Infectious Bovine Rhinotracheitis virus (IBRV). These four reproductive pathogens have been suspected to be present in dairy cattle in T&T for some time but, previously, studies have not been carried out to confirm their presence. Bulk milk samples were collected from 92 dairy farms across Trinidad, representing a total of 1177 dairy cattle. Four dairy farms were selected for individual milk sampling to assess in-farm seroprevalence levels. Milk samples were tested for antibodies to the four pathogens by commercial ELISA kits. The overall farm seroprevalence was 62% for *N. caninium* and 23% for IBRV, and no antibodies were detected in any of the bulk milk samples for *B. abortus* or BVDV. Mixed infections for IBRV and *N. caninum* were common. Seroprevalence levels were between 8% and 65% for *N. caninum* and between 3% and 53% IBRV on the four individual farms. These results reveal the presence of IBRV and *N. caninum* for the first time on the island of Trinidad and importantly reveal no evidence for the circulation of BVDV or *B. abortus* in dairy cattle in Trinidad.

## 1. Introduction

The twin island republic of Trinidad and Tobago is the southernmost nation in the Caribbean archipelago, just 11 km off the northeastern coast of Venezuela. Dairy farming in Trinidad, the larger island in the Republic of Trinidad and Tobago (T&T), mainly involves small-scale (<30 cows) semi-intensive farms, utilising single milking machines or hand milking for the most part [1]. There are also a small number (<10) of larger state-run farms which contain 50–100 milking cows, some of which utilise multiple unit milking machines. In an agricultural census carried out in 2004, it was estimated that there were approximately 10,700 cattle and 9000 water buffalo in T&T. In recent years, however, these numbers have been estimated to have reduced to 1500 water buffalo [2] and approximately 5–6000 cattle (unpublished data—Nestlé Trinidad and Tobago Ltd., Valsayn, Trinidad and Tobago, Extension Services, 2018).

Fertility of dairy herds has declined throughout the world in recent years [3,4,5] and abortion is known to be a major cause of economic loss in both dairy and beef industries worldwide. [6]. Diseases of the reproductive system, cause reductions in fertility and the magnitude of their effect depends on the severity and timing of the condition [7,8,9] and the herd management practices [10]. To date, no studies have been published from T&T investigating the economic impact of reproductive losses, abortion and reduction in fertility on the dairy farming industry in the country. Problems related to sub-fertility and abortion in dairy cattle are frequently reported by farmers, but detailed investigations are seldom carried out, leading to no definitive diagnosis. A large ten-year bovine abortion study carried out in the Midwestern United States, demonstrated that approximately 30% of abortions were attributed to an infectious cause, with half of those due to bacterial pathogens and 10% due to viral pathogens, almost equally divided between IBRV and BVDV [11]. In a UK study, BVDV, IBRV, *Leptospira hardjo*, and mixed bacterial/fungal infections were identified most often in cases eliciting a definitive diagnosis [12].

Diseases, such as Infectious Bovine Rhinotracheitis (IBR), Bovine Viral Diarrhoea (BVD), Neosporosis, and Brucellosis, are well known for causing infertility, sub-fertility, and abortions in dairy cattle [11]. *N. caninum* is an obligate intracellular parasite that has been documented as a major cause of abortion in cattle worldwide [13]. No studies have been published on the prevalence on *N. caninum* in cattle in T&T and the wider Caribbean region, although three studies in Grenada revealed the presence of the parasite in sheep and goats [14] and dogs [15], but not in pigs [16]. A seroprevalence study conducted in Mexico revealed a N. caninum seroprevalence of 72% among cattle, with associated high rates of abortion [17]. *Brucella abortus* (*B. abortus*) has been previously identified to be present in T&T cattle and buffalo [18], although seroprevalence studies have not been reported in domestic cattle and buffalo in recent years. Unconfirmed reports, however, indicate that *B. abortus* is circulating in some buffalo populations in Trinidad. Additionally, the current status of *B. abortus* circulation in domestic cattle is unknown. As *B. abortus* is a zoonotic pathogen, it is critically important to know whether (or not) the bacteria is currently circulating in the dairy cattle population. 

There are no previous published reports of the presence of Infectious Bovine Rhinotracheitis virus (IBRV) and Bovine Viral Diarrhoea virus (BVDV) in T&T and the wider Caribbean region. These two above-mentioned viruses are known to be circulating in the USA and Canada [19,20], countries that have been used in the past to source cattle for export into Trinidad. There is, therefore, an as yet unknown risk that these two viruses have been brought into Trinidad within imported cattle. 

This study therefore set out to generate baseline data related to the circulation of four important reproductive pathogens (IBRV, BVDV, *B. abortus*, and *N. caninum*) in dairy cattle in T&T. 

## 2. Materials and Methods

### 2.1. Sampling Strategy and Sample Collection

A brochure was distributed to all of the farmers participating in the study, detailing relevant information on the aims and objectives of the project, including information of the four reproductive pathogens under study. Bulk milk sampling was opportunistic and took advantage of the fact that the majority of dairy farms in Trinidad sell their milk directly to the company Nestlé, who then process and distribute the milk mostly within T&T and the wider Caribbean region. 

**Figure 1:**
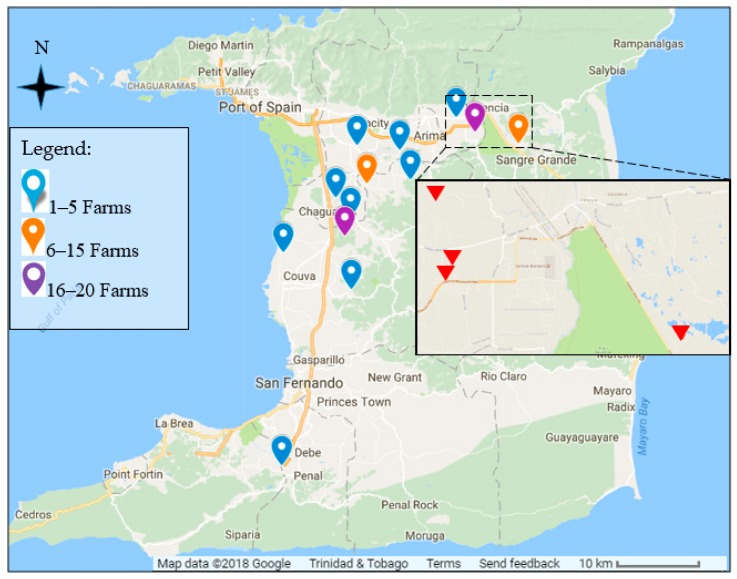
Map of Trinidad and Trinidad (10.6918° N, 61.2225° W) showing locations of dairy farms where bulk milk samples were collected. Inset: Wallerfield-Turure area showing locations of the four farms where individual milk samples were taken.

In total, bulk milk samples were collected from 92 dairy farms across the length and breadth of Trinidad, representing 1177 dairy cattle (Figure 1). Two sets of bulk milk samples (15 mL each) were collected from each of the 92 farms. The bulk milk samples (15 mL) were collected into Universal tubes each containing a potassium dichromate preservative tablet (Lactab Mark III, Thompson and Capper Ltd., Cheshire, UK). 

Four farms were selected for further individual milk sampling on the basis that they were antibody-positive in the bulk milk sampling for *N. caninum* and IBRV, and were located in a region of the country with a high density of dairy farms and frequent reports of subfertility and abortions. Eighty-five (85) milk samples were collected from all the milking dairy cattle in the four selected farms during the afternoon milking period. After the foremilk was removed, a sample of milk (15 mL) was collected manually into a universal tube containing a potassium dichromate preservative tablet (Lactab Mark III, Thompson and Capper Ltd., Cheshire, UK).

### 2.2. Serological Testing

All milk samples were tested for antibodies to IBRV, BVDV, *N. caninum*, and *B. abortus* by enzyme-linked immunosorbent assay (ELISA) using commercial test kits against the various pathogens (Table 1) following the manufacturer’s instructions. Specific details of the ELISA kits used, along with the sensitivities and specificities of the assays, are shown in Table 1. All ELISA tests were performed in duplicate as per the manufacturer’s instructions.

In the event of all samples testing negative for a particular infectious disease, the probability of having missed a positive sample in the total population was determined as 1 minus the probability that T&T is free of infection. Ausvet Freecalc [21] was used to estimate the probability of having missed a positive sample, taking into consideration the expected prevalence for that disease at a 95% confidence [22,23].

## 3. Results

Bulk milk samples from the 92 dairy farms were tested for antibodies to *B. abortus*, *N. caninum*, BVDV, and IBRV. All bulk milk samples tested negative for antibodies to *B. abortus* and BVDV. One bulk milk sample from the 92 farms tested low positive for *B. abortus* antibodies. This farm was subsequently revisited and all the individual cattle on the farm were sampled, with all samples testing negative for *B. abortus* antibodies. The probability of having missed a positive sample for BVDV and *B. abortus* in a population of 6000 cattle at an expected prevalence of 5% for BVDV and 15% for *B. abortus*, calculated using Ausvet FreeCalc (2018) with a 95% confidence, was 0.0467 and 0.05, respectively.

Nineteen (19) of the 92 dairy farms (20.7%) tested positive for antibodies to IBRV. Sixty-five (65) of the 92 farms (70.7%) tested positive for antibodies to *N. caninum.*

Individual milk samples were collected from all the milking cattle on four selected farms and were tested for antibodies to *B. abortus*, *N. caninum*, BVDV, and IBRV. All milk samples tested negative for antibodies to *B. abortus* and BVDV. The seroprevalence for IBRV ranged from 8–65% and the seroprevalence for *N. caninum* on the four farms ranged from 3–53% (Table 2).

## 4. Discussion

*B. abortus*, BVDV, IBRV, and *N. caninum* are responsible for causing major reproductive losses in dairy cattle worldwide [11,23,24]. Apart from *B. abortus,* which has previously been identified to be present in cattle and water buffalo in T&T [25], there have been no reports in the literature describing the presence of these pathogens in T&T. This study was, therefore, designed to specifically address whether any of these common reproductive pathogens were circulating in dairy cattle in T&T, and whether they were likely to be causing reproductive losses (abortion, infertility, and subfertility) as suspected. 

Brucellosis was first diagnosed in T&T in 1998, when a Holstein-cross cow that suffered a late-term abortion was found to be serologically positive. In later investigations, *B. abortus* was isolated from seropositive domestic cattle and water buffalo [18]. Between 1998 and 2001, a nationwide testing program was implemented during which time many seropositive cattle and water buffalo were sent to slaughter [25]. Studies have, however, continued to identify seropositive water buffalo in Trinidad [26]. There is a high risk that *B. abortus* may pass from water buffalo to dairy cattle, as both species often share grazing pastures and are often present on the same farms. *B. abortus* is an important zoonotic pathogen, so it is very important to know whether it is present (or not) in the domestic cattle population [27]. In the only study on the human population in Trinidad, 394 at-risk livestock/farm and abattoir workers all tested negative for *B. abortus* antibodies [28]. However, unpublished data identified the presence of three seropositive farm workers in 1998, when *B. abortus* cases were first discovered in cattle in Central Trinidad. To our knowledge, *B. abortus* has never been identified to be present in either cattle or humans on the sister island of Tobago. The result of this study, showing no evidence for *B. abortus* antibodies in the 92 dairy cattle farms that were sampled, is highly significant, as it indicates that *B. abortus* in unlikely to be circulating in the dairy cattle population of T&T. This important zoonotic pathogen is, therefore, currently likely to be confined to certain water buffalo populations in T&T, making its control and possible eradication from the country a viable prospect. These results also emphasise the importance of carrying out regular surveillance of the cattle and water buffalo populations in Trinidad for *B. abortus*, which could be carried out relatively easily through bulk milk sampling. 

This study reports the first recorded identification of cattle with antibodies to IBRV in the Caribbean, although there are several previous reports of IBR in Central and South America [29,30,31,32,33,34]. Identification of antibodies to IBRV indicates that the virus is circulating within the cattle population in T&T, so it is possible that this virus is causing cases of respiratory signs and reproductive problems observed in T&T cattle, which have so far gone undiagnosed. IBRV is transmitted horizontally between cows and by extension from farm to farm [35]. Consequently, given that there are many IBRV-negative farms present in T&T, which should work to maintain their negative status, the importance of maintaining good levels of biosecurity on farms and avoiding the movement of cattle from IBRV-infected to -free farms is of paramount importance. It has been shown that IBR spread to a previously uninfected farm can result in a 1 kg per cow per day decrease in milk production, resulting in decreased profitability [36]. This can be particularly devastating if the disease is introduced to the national herd [37]. 

The economic costs of BVDV infections worldwide vary markedly within, and between, countries [38]. In one year, BVD was estimated to cost between $760 million–$2.2 billion to producers in the USA [39]. For some time, farmers and vets in T&T have suspected that BVDV was present. However, the results from this study indicate that it is not present, which is good news for T&T, considering the seriousness and potential economic impact of the disease. BVDV has been documented to be present in dairy cattle in Uruguay, where 69% of cattle were seropositive [33], in Brazil [40], where a seroprevalence of 52% was demonstrated in the water buffalo and in Costa Rica, where seroprevalence levels of 19% and 27% were identified in cattle for BVD types 1 and 2, respectively [29]. 

*N. caninum* has been documented to be present in the English-speaking Caribbean, in Grenada, but only in dogs [15,41], sheep and goats [14], and pigs [16]. Although this is the first report of the presence of *N. caninum* in dairy cattle in T&T, serological evidence for the very similar *Toxoplasma gondii* has been reported for water buffalo [42], goats [43], and dogs [44]. With the known widespread and worldwide distribution of *N. caninum*, and its life cycle that includes dogs as definitive hosts, it is not surprising that the parasite is present in the cattle population of T&T. In many cattle farms in T&T, there is very close contact between cattle and farm dogs. The 65% seroprevalence level observed in cattle in this study is consistent with other studies from other countries [17,45]. It should also be noted that vertical, rather than horizontal, transmission between cattle is considered to be a major route of transmission, in one study being estimated at 44% [46].

It is important to note that seropositivity alone does not necessarily correlate with reproductive problems in all situations [47]. Identification of the actual pathogen itself in the aborting dam and/or in the aborted foetus or placental tissues is the best way of confirming that the pathogen is responsible for the abortion. Ideally, the routine investigation of all bovine abortion cases in a country should be carried out; however, this is neither a legal requirement nor a reality in the majority of Caribbean countries, including T&T. Even in countries with legislated bovine abortion reporting schemes, compliance is less than ideal [48]. The relatively high number of undetermined cases, 67% of nearly 9000 cases in one large study [11], possibly explains the reluctance to invest time and money into trying to gain a definitive diagnosis. The results provided in this study will hopefully be used to generate a standardized policy for the reporting of abortions in dairy cattle in T&T.

## 5. Conclusions

This study highlights the importance of regular serological monitoring of dairy cattle for economically important reproductive pathogens. Although antibodies to BVDV and *B. abortus* were not observed in T&T dairy cattle in this study, continued monitoring for these viruses is paramount, as outbreaks are being reported in neighbouring South American countries, and *B. abortus* is actively circulating in water buffalo in Trinidad. The presence of IBRV in 23% of dairy cattle farms in Trinidad emphasizes the need for uninfected farms to remain free of the virus through heightened biosecurity and movement restrictions. The high farm prevalence observed for *N. caninum* (62%) is not surprising, as this parasite is present in cattle in most countries around the world. Breaking the life cycle of the parasite, however, through reducing contact between dogs and cattle, especially at calving times, is strongly recommended as a control and prevention measure.

## Figures and Tables

**Table 1:** ELISA kits used for the detection of antibodies against four bovine infectious agents.

Infectious Agent	Test Kit	Sensitivity (%)	Specificity (%)	Company/Manufacturer
Infectious Bovine Rhinotracheitis virus (IBRV)	LSIVet Bovine IBR Screening Advanced—Milk	100	100	LSIVet^TM^
Bovine Viral Diarrhoea Virus (BVDV)	LSIVet Ruminant BVD/BD p80—Serum/Milk	97.3	97.9	LSIVet^TM^
*Neospora caninum*	LSIVet Bovine Neosporosis Advanced—Serum/Milk	100	98.5	LSIVet^TM^
*Brucella abortus*	IDEXX Brucellosis Milk—Bovine	100	95	IDEXX

**Table 2:** Percentage of dairy cattle positive for antibodies to IBRV and *Neospora caninum*.

	Number of Cattle	IBRV—% Positive (No.)	*N. caninum*—% Positive (No.)
Farm 1	17	65 (11)	53 (9)
Farm 2	39	8 (3)	3 (1)
Farm 3	15	53 (8)	40 (6)
Farm 4	17	18 (3)	29 (5)
